# Oberbrunnen, a New Mineral Water

**Published:** 1888-01-14

**Authors:** 


### OBERBRUNNEN, A NEW MINERAL WATER.

MANY people are inclined to say of mineral waters, what students say of articles in the Materia Medica, that there are at least ten times too many of them. The young man who has yet to face the examiners, looks upon an addition to the Pharmacopœia with quite different eyes from him whose experience has shown him that, in spite of the twelve hundred and more different drugs now in use, there is ample room for twelve hundred more, provided they be of the right kind. The same may be said of mineral waters, and indeed of every other article of consumption known to commerce; let them be but good and useful, and then the more the better. The proprietors claim for Oberbrunnen water that it is "an alkaline saline of a chemical composition, quite special and peculiar." That is an assertion easily proved or disproved, and as the water has been competently analysed, readers need not doubt that its chemical composition is "quite special and peculiar."

The chief constituents of the Oberbrunnen are the "bicarbonates of lithia, soda, and iron, the sulphate of soda, and free carbonic acid gas." Any intelligent medical man knows the value of a fluid of such a constitution, provided the proportions are suitable. In the case of the Oberbrunnen water the proportions are very suitable for a considerable variety of cases, and for practically all kinds of "no-cases," by which we mean persons in average health. Men, women, and children suffering from gout and its cousins and descendants, from liver derangements, from catarrhal affections of the throat or intestine, from anæmia and scrofula, are advised to try the new water, and the advice is undoubtedly good. Of course it does not, like certain well-known pills, profess to cure everything. If it did we would not have anything to say of it in any way. But it may be taken for granted that in many of the disorders indicated a prolonged course of the water will be of great service. It is decidedly both alterative and tonic, as well as very slightly aperient in large quantities.

The Oberbrunnen may be freely used as a table water, for which its agreeable taste and the "sharp" character imparted to it by the quantity of carbonic acid gas with which it is charged render it very suitable. There is not the least flavour or odour of "physic" about it. Its richness in the salts of lithia and soda make it peculiarly suitable for those who have gouty and rheumatic tendencies; whilst the iron which it contains gives it additional value. This water might usefully find a place in every household.

